# The Regulatory Role of Ferric Uptake Regulator (Fur) during Anaerobic Respiration of *Shewanella piezotolerans* WP3

**DOI:** 10.1371/journal.pone.0075588

**Published:** 2013-10-04

**Authors:** Xin-Wei Yang, Ying He, Jun Xu, Xiang Xiao, Feng-Ping Wang

**Affiliations:** 1 State Key Laboratory of Microbial Metabolism and School of Life Sciences and Biotechnology, State Key Laboratory of Ocean Engineering, Shanghai Jiao Tong University, Shanghai, PR China; 2 Key Laboratory of Systems Biomedicine, Ministry of Education, Shanghai Jiao Tong University, Shanghai, PR China; Instituto de Tecnologia Quimica e Biologica, Portugal

## Abstract

Ferric uptake regulator (Fur) is a global regulator that controls bacterial iron homeostasis. In this study, a *fur* deletion mutant of the deep-sea bacterium *Shewanella piezotolerans* WP3 was constructed. Physiological studies revealed that the growth rate of this mutant under aerobic conditions was only slightly lower than that of wild type (WT), but severe growth defects were observed under anaerobic conditions when different electron acceptors (EAs) were provided. Comparative transcriptomic analysis demonstrated that Fur is involved not only in classical iron homeostasis but also in anaerobic respiration. Fur exerted pleiotropic effects on the regulation of anaerobic respiration by controlling anaerobic electron transport, the heme biosynthesis system, and the cytochrome *c* maturation system. Biochemical assays demonstrated that levels of *c*-type cytochromes were lower in the *fur* mutant, consistent with the transcriptional profiling. Transcriptomic analysis and electrophoretic mobility shift assays revealed a primary regulation network for Fur in WP3. These results suggest that Fur may act as a sensor for anoxic conditions to trigger and influence the anaerobic respiratory system.

## Introduction

Iron is one of the most important micronutrients for bacterial growth and an essential cofactor for several proteins that participate in major cellular processes [1]. Due to the scarcity of available iron under aerobic conditions, as well as the toxicity of free iron at elevated concentrations via the Fenton reaction [2], bacteria employ a number of strategies by which to regulate intracellular iron concentrations, such as the synthesis and export of chelators [3], reduction by ferric reductases [4], and the expression of oxidative stress genes [5].

In most bacterial species, iron homeostasis is controlled by the ferric uptake regulator (Fur). Generally, Fur can act as both a positive and negative regulator of transcription. Fur senses excess intracellular Fe^2+^ and binds to the promoter regions of genes that participate in cellular processes, thereby directly obstructing or activating the transcription of these genes [6]–[8]. Even in its iron-free (apo) form, Fur can act as a transcriptional repressor [9]. Most indirect Fur regulation occurs at the posttranscriptional level in the presence of iron through the repression of a non-coding regulatory RNA (*ryhB*), to allow its target genes to be expressed [10]–[12]. In addition to its major role in the regulation of gene expression in the iron homeostasis system, Fur also functions as a pleiotropic transcriptional regulator and is involved in the control of diverse cellular processes, such as acid tolerance, redox-stress responses, flagellar chemotaxis, and virulence factor production [13]–[16]. Recent studies on the effects of iron concentration or *fur* inactivation have provided some evidence for the regulation of anaerobic respiration by Fur/iron in different bacterial species. In *Salmonella enterica,* nitrate respiration is controlled by Fur through the regulators NarP and NarL [17]. In *Shewanella oneidensis* MR-1, a *fur* mutation results in the reduced expression of genes encoding proteins that are involved in electron transport and cytochrome systems, such as *cymA* (tetraheme cytochrome *c*), *omcA/B* (decaheme cytochrome *c*), and *ccmH/E* (cytochrome *c* biogenesis protein), under anaerobic conditions [18]. In *Bacillus subtilis*, several cytochrome systems (e.g., *cydABCD)* have been reported to be repressed by iron limitation [19], and in *Pasteurella multocida,* the expression of genes that are involved in energy metabolism and electron transport (e.g., fumarate reductase, dimethyl sulfoxide reductase, and NapF) is decreased in response to iron restriction [20]. Moreover, FurA can act as a heme sensor protein [21] and directly control the tetrapyrrole biosynthesis pathway, which is involved in many metabolic processes, including anaerobic respiration, in *Anabaena* sp. PCC 7120 [22]. These studies have indicated a close relationship between iron regulation (primarily by Fur) and anaerobic respiration.

The *Shewanella* genus is known for its ability to use a broad range of electron acceptors, such as Fe (III), Mn (IV), trimethylamine *N*-oxide (TMAO), dimethyl sulfoxide (DMSO), sulfur, nitrate, and fumarate [23]. Members of this genus are ubiquitous in many environments and have been proposed as candidates for the bioremediation of metal and organic contaminants [24]. The majority of isolated *Shewanella* species are capable of iron respiration, in which iron plays essential roles as a both a protein cofactor and an EA [25]. Furthermore, the electron transfer chain in anaerobic respiration in *Shewanella* is predominantly composed of cytochrome *c* proteins, which contain heme groups that use iron as a cofactor [25], [26], indicating a relationship between iron regulation by Fur and anaerobic respiration in *Shewanella*.

The Fur protein is well conserved in the *Shewanella* genus [27]. In the model *Shewanella* species *Shewanella oneidensis* MR-1 (hereafter abbreviated as MR-1), which was originally isolated from Oneida Lake [28], Fur has been suggested to coordinate the regulation of energy metabolism. This conclusion was reached because mutations in *fur* in MR-1 affected the transcription of several genes that are involved in the electron transport system, energy metabolism, and regulation [15], [18], [29]. However, only a small number of physiological studies have compared the WT and *fur* mutant of MR-1, and these studies did not reveal any substantial differences in the growth or utilization of different EAs under anaerobic conditions [15], [18], [29]. Consequently, the role of Fur in the anaerobic respiration of *Shewanella* remains elusive.

Here, the role of Fur in anaerobic respiration was investigated in *Shewanella piezotolerans* WP3 (hereafter abbreviated as WP3), which was isolated from deep-sea sediments of the west Pacific [23], [30]. Deep-sea sediments contain high levels of authigenic ferric oxides [31] and low levels of oxygen. WP3 can use various external EAs under anaerobic conditions [32], and it is able to reduce hydrous ferric oxide to produce superparamagnetic magnetite particles with an average grain size of 4–6 nm [33]. The WP3 genome includes 55 putative cytochrome *c* genes, explaining the versatile respiratory capabilities of this strain [23].

To investigate the role of Fur in anaerobic respiration of the deep-sea bacterium WP3, a comparative transcriptomic analysis of WT WP3 and its *fur* mutant under anaerobic conditions was performed. In addition, physiological studies, cytochrome *c* content measurements, and DNA binding experiments were performed to verify the role of Fur in anaerobic respiration. Fur is shown to have important roles in regulating anaerobic respiration in WP3. This work calls for more attention on elucidating the general roles and molecular mechanisms of Fur regulation in deep-sea bacteria.

## Materials and Methods

### Bacterial Strains, Culture Conditions, and Physiological Studies

All bacterial strains and plasmids used in the present study are listed in Table S1. Cultures of *Escherichia coli* (*E. coli*) were grown aerobically in Luria–Bertani medium at 37°C. The *Shewanella* strains were cultured at 20°C under aerobic and anaerobic conditions. For aerobic cultivation, a modified 2216E culture (5 g tryptone, 1 g yeast extract, 34 g sodium chloride, and 50 mg FePO_4_ per liter) was used; for anaerobic cultivation, an oligotrophic medium (0.1 g tryptone, 0.2 g yeast extract, 34 g sodium chloride, 4.8 g HEPES, and 3.4 ml sodium lactate per liter) was dispensed into serum bottles gassed with O_2_-free nitrogen. After the media were autoclaved, the EAs were added at the required concentrations (2 mM nitrate; 20 mM dimethyl sulfoxide (DMSO); 20 mM fumarate, and 15 mM hydrous ferric oxide (HFO)) [34]. Chloramphenicol (25 µg ml^−1^ for *E. coli*; 12.5 µg ml^−1^ for WP3) was added to the medium when required. Siderophores were detected under anaerobic conditions (Coy anaerobic glove box) on solid culture medium via the application of chrome azurol-S (CAS)-based techniques. CAS screening plates were prepared using a previously described procedure [35], [36]. The HFO solution was prepared according to a previously described procedure [37]. The Fe^2+^ concentration was determined by measuring the absorbance at 562 nm on a SHIMADZU UV-2550 spectrophotometer (SHIMADZU CO., Kyoto, Japan) following the ferrozine method [33] after extraction with 1 N HCl. The OD_600_ was measured with a SHIMADZU UV-2550 spectrophotometer to obtain a growth curve. All of the physiological studies were performed in triplicate, and the average values and standard deviations were calculated.

### Generation of Mutant and Complementation Strains

The genes *fur* (Ferric uptake regulator), *ccmC* (cytochrome *c* biogenesis protein), and *fccA* (flavocytochrome *c*) were deleted in-frame using a fusion PCR method with the pRE112 plasmid, as previously described [38]. Chromosomal mutants were selected by resistance to chloramphenicol and sucrose, and deletions were confirmed using PCR sequencing.

For complementation, we used the *Shewanella–E. coli* shuttle plasmid vector pSW2, which was developed from the WP3 filamentous phage SW1 (unpublished data). The complete *fur* gene was ligated into the phage-based vector pSW2 to generate the pSW2-Fur plasmid. The plasmid was introduced into WM3064 by calcium chloride transformation and then mobilized into the *fur* mutant by mating. Complementation of the *fur* locus in the *fur* mutant strain was confirmed using PCR. The primers that were used to generate the PCR products are listed in Table S2.

### RNA Isolation and RNA Sequencing

Total RNA from WP3 WT and *fur* mutant strain at mid-log phase under anaerobic conditions using 20 mM fumarate as the EA were extracted in triplicate using Trizol reagent, respectively. The triplicate samples were mixed for RNA sequencing. Ribosomal RNA was removed using the RiboMinus™ Transcriptome Isolation Kit (Invitrogen, Carlsbad, CA, USA). RNA sequencing was performed on the Illumina HiSeq 2000 (Illumina, San Diego, CA, USA) at the Beijing Genomics Institute (BGI, China), following the manufacturer’s instructions. The accession code of our RNA-Seq dataset is GSE47773.

### RNA-seq Data Analysis and RT-PCR Validation

The raw sequencing data were trimmed of linker sequences, and a set of unique sequences was created by combining all of the reads with identical sequences. Unique sequences were mapped to the WP3 genome with SOAPaligner (soap2) [39]. The uniquely mapped reads were collected and analyzed with the DEG-seq package [40] to identify differentially expressed genes (estimation of gene expression based on RPKM values). The results of this analysis yield P- and Q-values for each gene to denote the difference in its expression between libraries [40]. In order to validate the data generated by RNA-seq, the expression levels of 8 randomly selected genes (swp0429, swp1175, swp1055, swp3209, swp3979, swp3980, swp3981 and swp4950) were quantified using RT-PCR. The RT-PCR log2 ratio values were plotted against the RNA-seq data log2 values.

### Fur Box Analysis and Logo Graph

The Fur Box was analyzed using the web-based tool RegPredict, which is available at http://regpredict.lbl.gov [41]. The Fur Box was identified by searching the 5′ regions of the genes in WP3 using the MR-1 information matrix for Fur. The information matrix for the generation of the Fur Logo was produced by aligning the WP3 Fur binding sequences predicted by the RegPredict web server. A graphical representation of the matrix through a Logo graph was obtained with Weblogo software, which is available at http://weblogo.berkeley.edu.

### RT-PCR Analysis

RT-PCR was performed using 7500 System SDS software and 20 µl reaction mixtures containing 10 µl SYBR Green-I Universal PCR Master Mix (Applied Biosystems, Warrington, UK), 0.5 µM of each primer, and 1 µl cDNA template. The primer pairs for the selected genes were designed using Primer Express software (Applied Biosystems, Foster City, CA, USA) (Table S2). In this method, *pepN*, which exhibits stable expression under various conditions, was used as the reference gene. The gene transcription levels of the targets were normalized to *pepN* in both the WT and *fur* mutant WP3 strains under anaerobic conditions [23]. RT-PCR assays were performed in triplicate for each sample. The mean value and standard deviation of the relative RNA expression levels were calculated.

### Cytochrome *c* Content Measurement

The WP3 WT and *fur* mutant strain were incubated at 20°C under aerobic and anaerobic conditions using 20 mM fumarate as the EA. The cells were harvested at mid-log phase by centrifugation and resuspended in the phosphate buffered saline (PBS). After the cells were lysed with an ultrasonic cell disruptor, the soluble protein was measured by Bradford protein assay. For equal part (50 µg), reduce the heme iron from Fe^3+^ to Fe^2+^ by adding a few grains of sodium dithionite to the sample, cover, and mix by slowly inverting until a color change upon reduction of the sample was observed. The cytochrome *c* content was evaluated in a spectrophotometer (Amersham Ultrospec 3100, GE Healthcare, USA), recording from 400 to 600 nm using the untreated protein as blank.

### Expression and Purification of the Fur Protein

The entire *fur* ORF (a 429-bp DNA fragment) containing an EcoRI site (5′-end) and an XholI site (3′-end) was PCR-amplified and then cloned into the EcoRI/XholI sites of the plasmid pET28a which carries an N-terminal His-tag. The resulting *fur* recombinant expression plasmid, pET28a-fur, was transformed into *E. coli* BL21 (DE3) cells. The cell cultures were incubated at 37°C in LB medium until an OD_600_ of 1.0 was reached. Protein expression was then induced by adding 0.1 mM IPTG (final concentration), and the cells were subsequently grown at 37°C for 4 h. The cells were then harvested by centrifugation and resuspended in 20 ml PBS. After the cells were lysed with an ultrasonic cell disruptor, the cell lysate was purified using a nickel-nitrilotriacetic acid (Ni-NTA) agarose column as directed by the manufacturer (GE Healthcare). Recombinant Fur was eluted with elution buffer containing 500 mM imidazole.

Purified Fur from the elution buffer was concentrated in the phosphate buffered saline using Amicon Ultra-15 Centrifugal Filter Unit with Ultracel-10 membrane, according to the manufacturer’s protocol (Millipore Corporation, Bedford, MA). The concentration of the protein was determined by the Bradford assay.

### Electrophoretic Mobility Shift Assay (EMSA)

Double-stranded DNA probes were generated by PCR with DIG-labeled dNTPs using the primers listed in Table S2 and purified with the Cycle Pure Kit (Omega). The binding reaction was performed with ∼0.2 pmol DIG-labeled probes and ∼200 pmol purified Fur protein in 20 µl binding buffer containing 40 mM KCl, 12.5 mM Tris (pH 7.5), 125 µM MnCl_2_, 1.25 mM MgCl_2_, 5% glycerol, 0.5 mM DTT, 0.01% BSA and 50 µg/ml Salmon Sperm DNA. Specific competitors (2 pmol and 20 pmol unlabeled probes) were added when necessary. The reaction mixtures were incubated at 20°C for 30 min and then loaded onto 6% non-denaturing polyacrylamide gels. Following non-denaturing polyacrylamide gel electrophoresis, gel was transferred onto positively charged nylon membrane (Amersham, GE Healthcare, USA), and UV-cross linked. The membrane was then subjected to detection by chemiluminescent EMSA kit (Pierce, Thermo Scientific, USA) following the manufacturer’s protocol.

## Results and Discussion

### Generation and Physiological Evaluation of the WP3 *fur* Mutant

**Figure 1:**
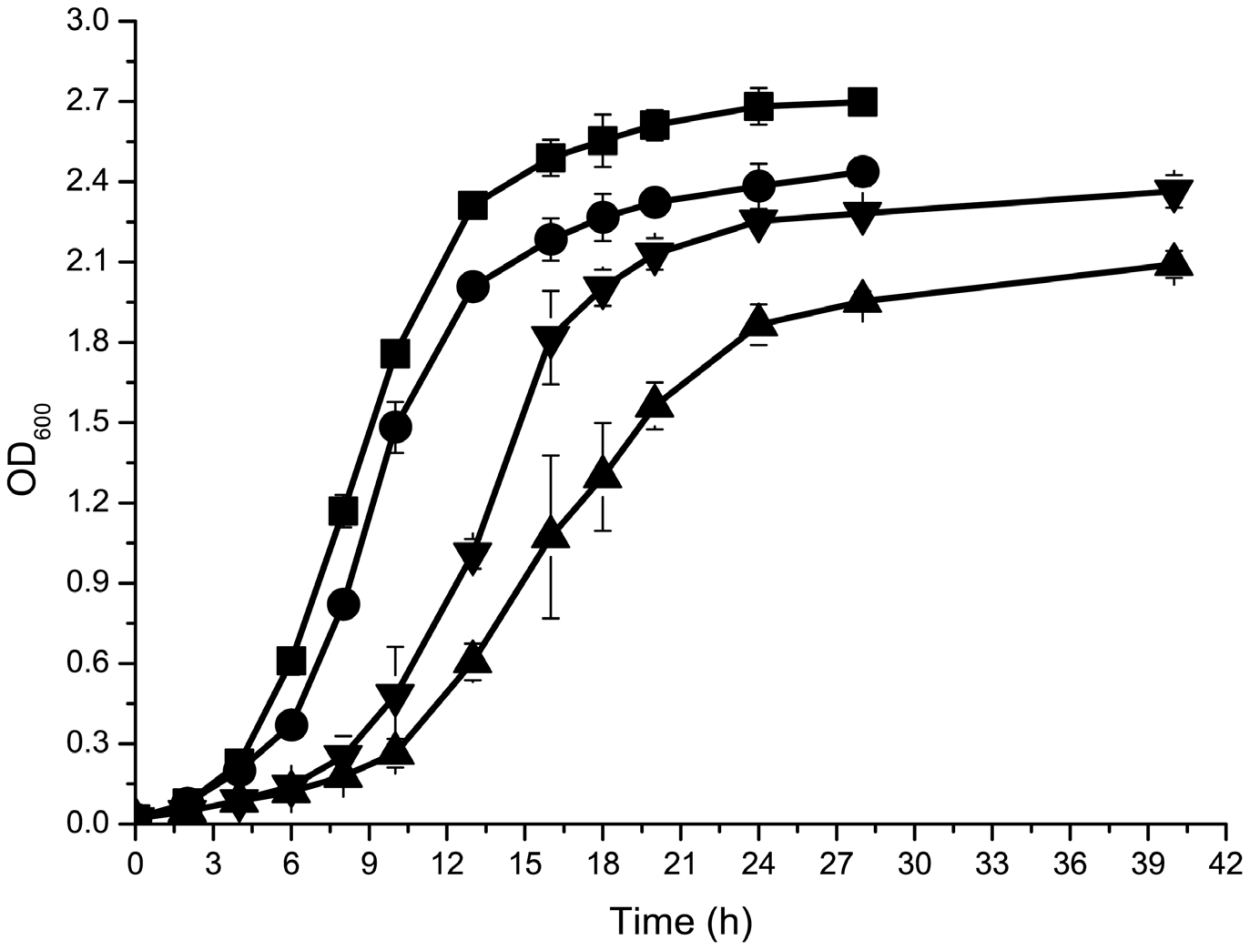
Growth curves of WT WP3 and the *fur* mutant in liquid 2216E with or without 60 µM iron chelator under aerobic conditions. (▪) WT WP3, (•) *fur* mutant, (▴) WT WP3 with 60 µM iron chelator, and (▾) *fur* mutant with 60 µM iron chelator. The data represent averages of triplicate cultures.

A *fur* deletion mutant was constructed in WT WP3. When grown aerobically at 20°C on 2216E agar medium, the *fur* mutant formed smaller colonies than the WT strain. Similarly, the mutant exhibited a lower growth rate than the WT strain when cultivated in liquid 2216E medium (Figure 1), indicating that *fur* inactivation resulted in a slight growth deficiency under aerobic conditions. The colonies of the *fur* mutant also appeared paler in color than WT WP3 colonies, potentially indicating the presence of lower heme levels because the pink pigmentation of WT WP3 cells has been attributed to high heme content [26]. To further investigate the behavior of the *fur* mutant, an iron chelator (2, 2′-dipyridyl) was added to the liquid 2216E medium to mimic iron depletion. In the presence of 60 µM iron chelator, both strains displayed clear growth inhibition. Notably, the *fur* mutant displayed a much shorter lag phase than the WT strain and achieved a higher cell density at stationary phase (Figure 1), suggesting that the *fur* mutant had a higher tolerance to the stress of iron depletion, consistent with the findings in MR-1 [15]. Anaerobic incubations on CAS screening plates revealed that the *fur* mutant produced a larger yellow halo around the colony periphery than the WT strain, indicating that the *fur* mutant possessed an enhanced ability to produce a diffusible, Fe(III)-chelating compound that outcompeted the WT strain for Fe(III) (Figure S1). This enhancement also explains the better growth of the *fur* mutant under iron-depleted conditions compared to the WT strain.

**Figure 2:**
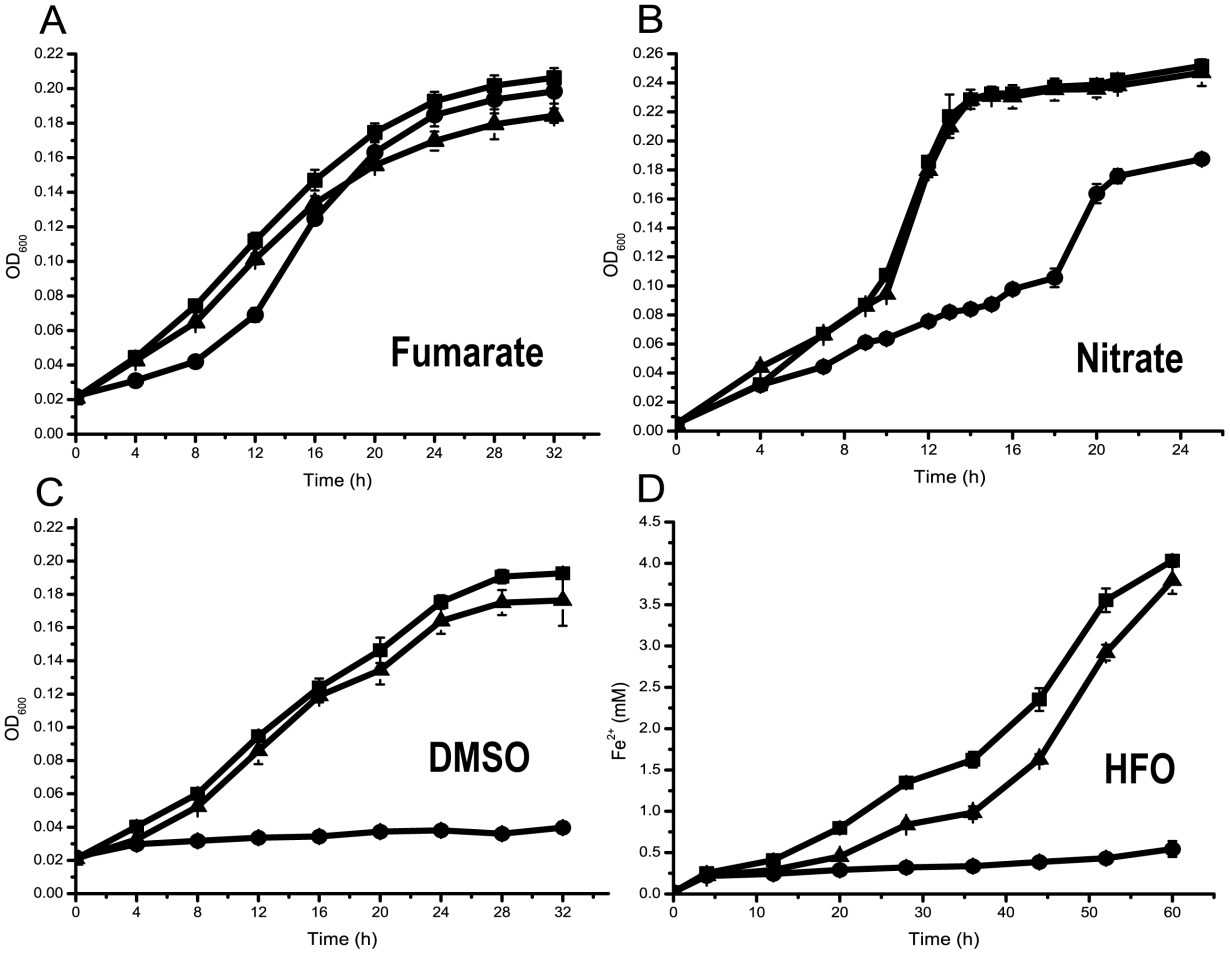
Growth curves of the WT WP3, *fur* mutant, and *fur* complement strains with different electron acceptors under anaerobic conditions. (**a–c**) Growth on 20 mM fumarate, 2 mM nitrate, and 20 mM DMSO as the electron acceptor, respectively. (**d**) Time course of Fe^2+^ concentration with 15 mM HFO as an electron acceptor. The following abbreviations are used for all of the panels: (▪) WT WP3, (•) *fur* mutant, (▴) *fur* complement strain. The data represent averages of triplicate cultures.

To elucidate the roles of Fur related to the anaerobic respiration in WP3 cells, the *fur* mutant WP3Δ*fur* was cultivated in oligotrophic medium with fumarate, nitrate, DMSO, or HFO as the sole EA. The initial growth rate of the *fur* mutant on fumarate was lower than that of the WT strain; however, the growth rates of the strains were nearly identical at stationary phase (Figure 2A). In the presence of nitrate, the *fur* mutant exhibited a pronounced lag phase in growth, with a much lower growth rate and lower cell densities compared to the WT strain (Figure 2B). The most significant growth deficiency was observed for the mutant grown in the presence of DMSO (Figure 2C), and the initial reduction in HFO was decreased severely (Figure 2D). To confirm the casual relationship between the disruption of the *fur* gene and the differences in the growth of the *fur* mutant and the WT strain, a complementation assay was conducted by cloning and introducing the *fur* gene back into the mutant strain. As shown in Figure 2, the recovery of the *fur* gene in the mutant restored its respiratory ability when any of the EAs were provided. These results confirmed the role of Fur in the regulation of respiration, particularly in the anaerobic respiration of WP3 cells.

Previous physiological studies of MR-1 demonstrated that the *fur* mutant resembled the parental strain in its ability to grow anaerobically on different EAs such as MnO_2_, Fe(OH)_3_, Fe(III) citrate, nitrate, nitrite, DMSO, TMAO, fumarate, thiosulfate, sulfite, and AQDS [29]. Recently, the growth rate of the *fur* mutant was also tested in *Salmonella enterica* serovar Typhimurium, *Dichelobacter nodosus*, and *Desulfovibrio vulgaris*; in these strains, the absence of *fur* did not cause any notable changes in growth under anaerobic conditions [42]–[44]. Here, a series of physiological experiments confirmed the roles of Fur in the regulation of anaerobic respiration in WP3. Iron is a cofactor of heme, an important component of cytochromes for electron delivery during the anaerobic respiration of *Shewanella* [45]. Fur is a major regulator in iron homeostasis, and it is thus likely that Fur is utilized in the regulation of the anaerobic respiration system. However, the mechanism by which Fur exerts its influence in anaerobic respiration remains to be elucidated.

**Figure 3:**
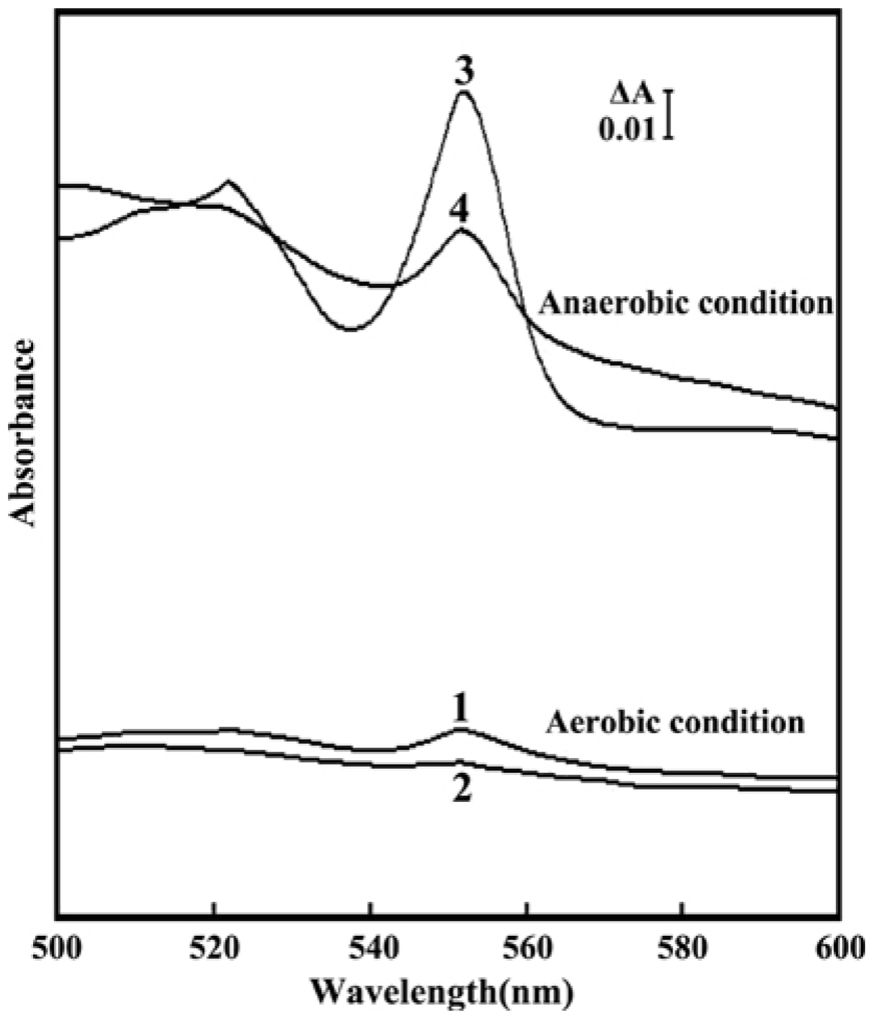
Spectrum analysis of the cytochrome *c* content of WT and *fur* mutant strains under aerobic and anaerobic conditions. The reduced-minus-oxidized difference spectra of equal amounts of total protein from the WT and *fur* mutant strains treated with sodium dithionite were recorded. The absorbance levels of the corresponding untreated strains were set as a control. **Line 1** Absorbance of the aerobically grown WT strain. **Line 2** Absorbance of the aerobically grown *fur* mutant strain. **Line 3** Absorbance of the anaerobically grown WT strain. **Line 4** Absorbance of the anaerobically grown *fur* mutant strain.

All sequenced *Shewanella* genomes include a large number of *c*-type cytochrome genes; for example, 55 *c*-type cytochrome-encoding genes were detected in WP3 [23], and the products of these genes are believed to transfer electrons to EAs [46]. To assess the impact of Fur on the cellular levels of *c*-type cytochromes, reduced-minus-oxidized difference spectra were obtained (Figure 3). The absorption maxima peak of *c*-type cytochromes occurred at ≈550 nm. The results revealed that (1) the amount of *c*-type cytochromes in each strain was higher under anaerobic conditions than aerobic conditions and (2) the *fur* mutant contained greatly reduced levels of *c*-type cytochromes compared to the WT strain, particularly under anaerobic conditions. These results suggest that the Fur protein might regulate anaerobic respiration by affecting the levels of *c*-type cytochromes.

### Genes Regulated by Fur

To examine the global impact of Fur in anaerobic respiration, comparative transcriptomic analysis was performed for the WP3Δ*fur* and WT WP3 strains. The data generated using RNA-seq were validated by quantitative PCR, and a high correlation (r^2^ = 0.839, n = 8) was observed between the two transcriptional datasets.

In total, 1160 genes (approximately 23% of the WP3 genome) exhibited differential expression under anaerobic conditions (at least 2-fold difference), and the COG annotations of these genes are displayed in Table 1. The absence of *fur* resulted in the increased expression of 988 genes and the decreased expression of 172 genes, indicating that Fur acts primarily as a repressor in the global regulation in WP3.

**Table 1:** Number of differentially expressed genes in Δ*fur*.

Differentially Expressed Genes in Δ*fur*			
Cluster of Orthologous Groups	Number of “Fur Repressed”^a^ Genes	Number of “Fur Activated”^b^ Genes	Total
No COG Assigned	174	57	231
Energy production and conversion (C)	76	14	90
Cell cycle control, cell division, and chromosome partitioning (D)	7	1	8
Amino acid transport and metabolism (E)	71	6	77
Nucleotide transport and metabolism (F)	27	1	28
Carbohydrate transport and metabolism (G)	20	1	21
Coenzyme transport and metabolism (H)	48	7	55
Lipid transport and metabolism (I)	33	4	37
Translation, ribosomal structure, and biogenesis (J)	99	5	104
Transcription (K)	39	27	66
Replication, recombination, and repair	43	1	44
Cell wall/membrane/envelope biogenesis (M)	60	4	64
Cell motility (N)	25	3	28
Posttranslational modification, protein turnover, and chaperones (O)	40	5	45
Inorganic ion transport and metabolism (P)	54	9	63
Secondary metabolites biosynthesis, transport, and catabolism (Q)	14	0	14
General function prediction only (R)	72	12	84
Function unknown (S)	62	10	72
Signal transduction mechanisms (T)	34	13	47
Intracellular trafficking, secretion, and vesicular transport (U)	49	3	52
Defense mechanisms (V)	17	3	20
Total	1064	186	1250

Categorized According to Cluster of Orthologous Groups (COGs).

a Genes with increased expression in the absence of *fur.*

b Genes with decreased expression in the absence of *fur.*

Among the genes with defined functions (Table 1), the following two groups were highly enriched with differentially expressed products: (1) genes involved in translation, ribosomal structure and biogenesis (2) genes related to energy production and conversion. Interestingly, the expression of genes encoding polar flagellum (swimming) and phages were also induced significantly in the *fur* mutant, indicating the role of Fur in motility and phage formation. Furthermore, 101 genes in 53 operons were identified having putative Fur binding sites in their corresponding upstream promoter regions (Table S3). The putative element with a 10-1-10 inverted repeat in WP3 (Figure S2) showed high sequence identity to the consensus sequence of MR-1 [18].

#### (1) Genes with functions in iron homeostasis

The largest gene module identified was composed nearly exclusively of an iron acquisition system, in agreement with the crucial role of Fur in iron homeostasis (Table 2). This result is also consistent with the results of the CAS-based analysis, which indicated increased iron absorption in the *fur* mutant under anaerobic conditions. Four homologous TonB systems have been annotated in the WP3 genome (swp3077–3080, swp3204–3207, swp3979–3981, and swp4948–4954). In gram-negative bacteria, TonB systems utilize the proton motive force across the cytoplasmic membrane to transduce the energy for delivering iron-siderophore complexes into the periplasmic space [47]. In the *fur* mutant WP3Δ*fur*, the TonB1 (swp3979–3981) and TonB2 (swp4948–4954) transporting systems were significantly induced, while there were much smaller or no detectable changes in the mRNA levels of the other systems (TonB3 and TonB4 transport systems). Notably, Fur Box motifs were identified upstream of the two TonB operons but not upstream of the other two operons (Table S3). The differences in the expression and gene regulation of the four TonB systems suggest that they may possess different functions in facilitating the uptake of various iron sources. Similar findings in *Vibrio* spp. and MR-1 have been reported [18], [48].

**Table 2:** Fur-responsive modules for iron acquisition and storage systems with fumarate as the EA.

Functional category	ORF		Gene product	WT/Δ *fur*log2 ratio	p-value	q-value
Ferredoxin	swp0771		Bacterioferritin-associated ferredoxin (Bfd)	−4.92	0	0
Ferrous iron transport system	swp3270		Ferrous iron transport protein B (FeoB)	−2.10	6.5E-131	2.4E-129
	swp3271	Ferrous iron transport protein A (FeoA)		−1.93	7.87E-37	9.78E-36
TonB-dependent receptor	swp0083		TonB-dependent siderophore receptor	−3.84	1.62E-18	1.14E-17
	swp3978	TonB-dependent Heme/hemoglobin receptor (HmuA)		−6.45	0	0
	swp5150	TonB-dependent siderophore receptor		−4.56	4.4E-277	3.6E-275
Energy-transducing TonB system	swp3979		TonB, C-terminal	−8.35	7.91E-37	9.79E-36
	swp3980	ExbB proton channel		−5.44	1.19E-44	1.78E-43
	swp3981	Biopolymer transport protein ExbD		−5.13	3.61E-25	3.13E-24
	swp4950	Biopolymer transport protein ExbD		−5.34	1.04E-93	2.95E-92
	swp4952	Biopolymer transport protein		−5.31	1.31E-60	2.52E-59
	swp4953	ExbB proton channel		−5.30	9.34E-61	1.81E-59
Siderophore biosynthesis system	swp0084		Siderophore biosynthesis protein	−6.34	3.01E-12	1.49E-11
ABC transporter system	swp3982		ABC hemin transporter (HmuB)	−5.27	1.32E-64	2.72E-63
	swp3983	ABC hemin transporter (HmuC)		−6.07	1.46E-32	1.64E-31
	swp3984	ABC hemin transporter (HmuD)		−6.18	8.24E-69	1.79E-67
	swp4105	ABC iron (III) transporter (FbpA)		−3.73	8.08E-17	5.16E-16
	swp4106	ABC iron (III) transporter (FbpB)		−3.16	2.5E-10	1.1E-09
	swp4107	ABC iron (III) transporter (FbpC)		−4.08	0	0
Iron storage system	swp0167	Ferritin and Dps		1.21	2.54E-07	8.82E-07
	swp1175	Bacterioferritin		4.00	9.82E-52	1.65E-50
	swp1176	Bacterioferritin		2.31	8.25E-18	5.59E-17

Interestingly, the iron storage proteins (ferritin and bacterioferritin) that displayed increased expression in the MR-1 *fur* mutant [18] displayed repressed expression patterns in the WP3 *fur* mutant constructed in the present study (Table 2). Iron storage systems can sequester excess iron, decrease iron toxicity, and decrease the production of reactive oxygen species (ROS) via the Fenton reaction [49]. In *E. coli*, Fur indirectly regulates intracellular iron storage by repressing the expression of a the small RNA RyhB in the presence of iron [50]. In this study, Fur Box motifs were identified upstream of genes implicated in iron storage (Table S3), suggesting that these genes are directly regulated by Fur in WP3. This result is in accordance with the direct positive regulation reported in *V. cholera*, *Neisseria meningitides*, and *E. coli* [6]–[8].

#### (2) Genes encoding secondary regulators

The indirect expression pattern suggested that Fur may act with other regulators to coordinate anaerobic respiration. Several secondary regulators or regulatory proteins were observed to be regulated by the Fur protein. Among these regulators, ArcA is a global regulator that controls hundreds of genes involved in aerobic/anaerobic respiration in a few gram-negative bacterial species [51]–[54]. The *arcA* mutant of MR-1 exhibits impaired aerobic growth and defective utilization of DMSO in the absence of O_2_ [53], [55], and ArcA was previously shown to be required for the regulation of cytochrome *c* proteins [54]. The change in *arcA* expression (∼4-fold increase in Δ*fur*) in WP3 suggests that Fur regulates anaerobic respiration indirectly through ArcA. The Crp/Fnr family transcriptional regulator swp3806, which is an ortholog of the *V. cholerae* cAMP-binding protein Crp (66% identity), was up-regulated (∼16-fold increase) in the *fur* mutant, with a Fur Box located upstream of the Crp-like regulator gene. Crp was previously reported to be a major regulator of anaerobic respiration; in MR-1, *crp* mutants are defective in using several EAs [56], [57]. It is very likely that Fur regulates anaerobic respiration in WP3 by interacting with the Crp-like protein directly, as there is a Fur Box in its promoter region. The TetR family transcriptional regulator SO1415 was characterized as a transcriptional factor that is involved in anaerobic energy metabolism in MR-1 [58]; its homolog in WP3, swp4152, displayed a ∼2-fold decrease in expression in the Δ*fur* mutant, suggesting that it may be a novel transcriptional factor in anaerobic respiration. A relationship was also observed between Fur and histone-like nucleoid structuring protein (H-NS) (swp3473, ∼3-fold increase in Δ*fur*). In *S. typhimurium,* Fur regulates HilA expression and virulence by negatively regulating H-NS [59]. In *E. coli*, Fur-mediated activation of *ftnA* transcription is due to Fur binding to the *ftnA* promoter region, resulting in competition for H-NS binding [7]. In WP3, Fur likely exerts its influence on anaerobic respiration at least partially through interaction with secondary regulators and regulatory proteins.

#### (3) Genes with functions in the anaerobic electron transport system

Eleven genes that are involved in anaerobic electron transport were significantly repressed in the *fur* mutant (Table 3); this finding may explain the deficient growth of the *fur* mutant under anaerobic conditions with a variety of EAs. The tetraheme *c* cytochrome CymA, a key protein that controls respiration in the presence of a variety of EAs, such as metals, DMSO, nitrate, and nitrite [60], [61], was repressed in the *fur* mutant. A *cymA* gene deletion mutant of WP3 (constructed in a previous study) displayed growth deficiencies when a variety of EAs were tested, including fumarate, HFO, DMSO, nitrate, and nitrite [34]. However, mutation of *cymA* only partially influenced fumarate respiration, indicating that other proteins are involved in receiving electrons from the menaquinone pool under fumarate-respiring conditions. No Fur-binding box motif was identified upstream of the *cymA* gene, suggesting potential indirect regulation by Fur.

**Table 3:** Fur-responsive modules for anaerobic electron transport with fumarate as the EA.

Functional category	ORF	Gene product	WT/Δ *fur*log2 ratio	p-value	q-value	
Anaerobic respiration system	swp4806	Nitrate/Nitrite/TMAO/metal/DMSO/Fumarate reductases, membrane-bound tetrahemecytochrome *c* (CymA)	1.25	2.18E-37	2.77E-36	
Anaerobic metal reduction system	swp3277	Decaheme cytochrome *c* (OcmA)	1.43	7.05E-29	6.96E-28	
	swp3278	Decaheme cytochrome *c* (MtrC)	2.44	2.8E-182	1.4E-180	
	swp3279	Decaheme cytochrome *c* (MtrA)	1.37	1.9E-55	3.47E-54	
	swp3280	Outer membrane protein precursor (MtrB)	1.19	2.44E-16	1.53E-15	
Anaerobic nitrate respiration system	swp4456	Periplasmic nitrate reductase (NapDβ)	2.04	1.1E-168	4.7E-167	
	swp4457	Anaerobic dehydrogenases (NapAβ)	1.37	4.56E-17	2.95E-16	
	swp4458	Nitrate reductase (NapBβ)	1.69	1.48E-07	3.8E-07	
	swp2772	Membrane-bound nitrate reductase (NapC)	−1.25	1.91E-13	1.01E-12	
	swp2773	Periplasmic nitrate reductase, (NapBα)	−1.81	1.19E-14	6.7E-14	
	swp2774	Anaerobic dehydrogenase (NapAα)	−2.34	4.14E-08	1.55E-07	
	swp2775	NapDα	−2.48	1.05E-15	6.34E-15	
Anaerobic nitrite respiration system	swp0613	Cytochrome *c* nitrite reductase (NrfA)	−2.01	8.87E-06	2.54E-05	
Anaerobic DMSO respiration system	swp0724	Twin-arginine translocation pathwaysignal (DmsA)	1.44	2.33E-06	7.19E-06	
	swp3456	Conserved hypothetical protein (DmsH)	1.80	0.000747	0.001609	
Anaerobic fumaraterespiration system	swp0220	Fumarate reductase, flavoprotein subunit	−3.74	1.36E-23	1.12E-22	
	swp0429	Fumarate reductase, transmembrane subunit	4.67	8.1E-193	4.3E-191	
	swp2950	Fumarate reductase, iron-sulfur protein subunit	−2.45	1.17E-27	1.12E-26	
	swp2952	Fumarate reductase, flavoprotein subunit	−2.49	1.11E-17	7.46E-17	
Others	swp4579	Cytochrome *c*, putative	−7.03	6.9E-211	4E-209	
	swp4577	Cytochrome *c* family protein	−6.57	0	0	
	swp4146	Cytochrome, putative	−2.42	8.38E-05	0.000208	
	swp3856	Cytochrome *c*	−3.12	7.58E-06	2.19E-05	

In addition to *cymA*, a variety of genes encoding *c*-type cytochromes were also regulated by Fur in WP3 (Table 3), including a periplasmic protein (MtrA), a cell-surface decaheme cytochrome *c* (MtrC/OmcA), and an integral outer-membrane protein (MtrB), which are all essential for metal reduction [46]. The repression of the *omcA*-*mtrABC* operon may have prevented the *fur* mutant from reducing HFO during the initial phase of growth. A Fur Box motif was identified within the upstream sequence in the putative promoter region of *omcA*, suggesting positive regulation through the direct binding of Fur.

There are two functional periplasmic dissimilatory (NAP) nitrate-reducing systems in WP3 (NAP-α and NAP-β), and deletion of either system has little effect on the ability of the cells to respire nitrate [34]. In this study, both of these NAP systems were regulated by Fur; the genes in the NAP-α system (swp2272–2275) were up-regulated in the *fur* mutant, while those of the NAP-β system (swp4456–4458) were down-regulated. Because our experiment was conducted at 20°C, the NAP-β system dominated the nitrate reduction. Moreover, a conserved Fur Box was identified upstream of *napD* (swp4456, NAP-β system). In conjunction with the growth deficiencies under nitrate-respiring conditions, these results indicate that Fur is involved in nitrate respiration. A similar involvement of Fur in nitrate/nitrite respiration was observed in *Salmonella enterica* [17].

The transcriptomic data revealed that a variety of putative fumarate reductase genes are differentially regulated by Fur. Among these genes, flavocytochrome *c* (swp4352) displayed the highest identity (63%) with the periplasmic fumarate reductase FccA in MR-1. FccA is the sole fumarate reductase given that the MR-1 FccA deletion mutant is unable to reduce fumarate [62]. Similarly, the *fccA* deletion mutant of WP3 did not grow under fumarate-respiring conditions. However, the expression of the flavocytochrome *c* was only slightly changed in WP3, indicating that other factors are involved in the clear physiological change at mid-log phase under fumarate-respiring conditions.

Overall, the results clearly demonstrate that Fur plays an important role in controlling the expression of genes that are involved in anaerobic electron transport. The identification of a conserved Fur Box motif in the promoter regions of the genes mentioned above suggests that these genes may be regulated through direct binding of Fur.

#### (4) Genes with functions in heme biosynthesis and transport

Heme is an iron-containing cofactor that is involved in redox reactions within cells [63]. Most heme-containing *c*-type cytochromes, such as CymA, NrfA, and MtrA, facilitate electron transport during anaerobic respiration [34], [46]. Heme can be obtained from external sources or produced by a dedicated biosynthetic pathway [63]. The expression of genes that are involved in heme biosynthesis, including *hemA* (swp3892), *hemB* (swp0440), *hemC* (swp0402), and *hemK* (swp0051 and swp4046), was significantly decreased in the *fur* mutant (Figure S3). The glutamyl tRNA reductase gene (*hemA*) is the first committed step in heme biosynthesis [64]. Meanwhile, putative genes for heme transport, such as the ABC-type heme transport system (hmuUTV, swp3982-swp3984) and the TonB-dependent heme/hemoglobin receptor (*hugA*, swp3978), were largely up-regulated (>20-fold, Table 2) in the *fur* mutant. The deficiency in heme biosynthesis was also reflected by the paler color of mutant cells. These data demonstrate that both the heme biosynthesis and electron transport systems are regulated by Fur, influencing the cytochrome *c* content and anaerobic respiration of cells.

#### (5) Genes with functions in the cytochrome *c* maturation system

The *c*-type cytochromes are ubiquitous hemoproteins that function primarily as electron carriers between enzymes involved in cellular energy transduction processes, such as photosynthesis and/or respiration [65]. In addition to its role in the maturation of *c*-type cytochromes, the cytochrome *c* maturation (CCM) system regulates cytochrome *c* content in bacterial cells [66]. The significant decrease in the *c*-type cytochrome content in the WP3 *fur* mutant indicates a relationship between the CCM system and the Fur regulation system. The complex CCM system is composed of ten components (CcmABCDEFGH, DsbA, and DsbD) and functions in transporting heme from the cytoplasm to the periplasm and to maturated apo-cytochrome *c* [66]. All of the CCM system genes were observed to be repressed in the *fur* mutant. To validate the transcriptomic results, RT-PCR assays were conducted to detect changes in expression levels (Figure S4). Among the CCM system genes, the *ccmC* gene displayed the most significant decrease in expression, by approximately 75% in the *fur* mutant compared to WT WP3. A *ccmC* gene deletion mutant was constructed to evaluate the potential role of the system in anaerobic respiration. The *ccmC* mutant cells exhibited a whitish color, similar to that of the *fur* mutant, and were unable to respire anaerobically when fumarate, Fe(III) and DMSO were provided as EAs (data not shown). These data suggest that repression of the CCM system may cause a significant loss in cytochrome *c* content in the *fur* mutant under anaerobic conditions.

### Experimental Validation of the Predicted Fur Box by EMSA

A Fur Box motif was identified upstream of several genes/operons involved in anaerobic respiration, such as *omcA*, *napD*, and the Crp-like regulator gene (Table S3). In MR-1, *omcA* also possesses a potential Fur-binding site in its upstream region [18]. The *omcA*, *napD*, and Crp-like regulator genes were all repressed in the *fur* mutant under fumarate-respiring conditions. To evaluate the functionality of the predicted Fur-binding sites, the regulatory regions of these three genes were subjected to PCR amplification and a gel mobility shift assay with the WP3-purified Fur protein. The WP3 Fur protein was purified as a recombinant His-tagged protein expressed in *E. coli*, and its activity was confirmed by binding to a known Fur Box in the TonB receptor promoter region. Non-Fur Box DNA fragment (swp1869 promoter) with a size similar to that of each of the investigated fur-binding regions was used as a negative controls (Figure S5).

**Figure 4:**
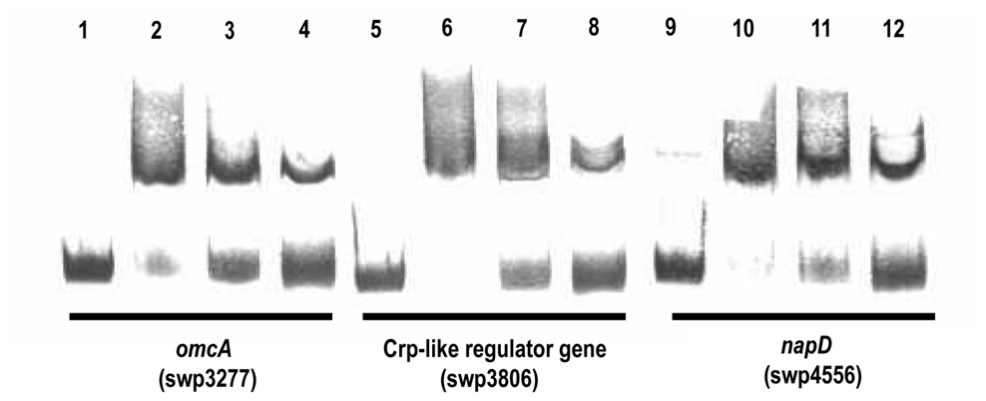
Fur binding to selected promoters (*omcA*, Crp-like regulator gene and *napD*) by EMSA. The binding assays were performed in the presence of 200(lanes 2–4, lanes 6–8, lanes 10–12) and 0.2 pmol DIG-labeled (lanes 1–12) promoter DNA. Non-specific competitor DNA (50 µg/ml Salmon Sperm DNA) was used in all these binding reactions to control for the presence of unspecific binding. Specific competitors (2 pmol and 20 pmol unlabeled probes) were added respectively in lane 3, 4; lane 7, 8; lane 11, 12 to verify the specificity of a band resulting from protein-binding to the labeled probe.

All three tested fragments were shifted in the presence of the purified Fur protein (Figure 4). The binding of Fur to the target promoters were not influenced by addition of the nonspecific competitor salmon sperm DNA, but were outcompeted by adding excess unlabeled probes (Figure 4). These results demonstrated the *in vitro* specific binding of Fur to all three of the DNA fragments with predicted Fur-binding sites. The interaction between Fur and the Crp-like regulator confirmed the hypothesis that Fur indirectly regulates anaerobic respiration through secondary regulators. Furthermore, the transcriptomic data showed that the *napD* operon, which is involved in nitrate reduction, and the *omcA* gene, which is involved in iron reduction, were both down-regulated in the *fur* mutant. Together with the confirmed Fur Box in the promoter regions, expression patterns of these two genes had indicated a Fur-dependent activation of each under anaerobic conditions.

### Fur Regulation Model in WP3

**Figure 5:**
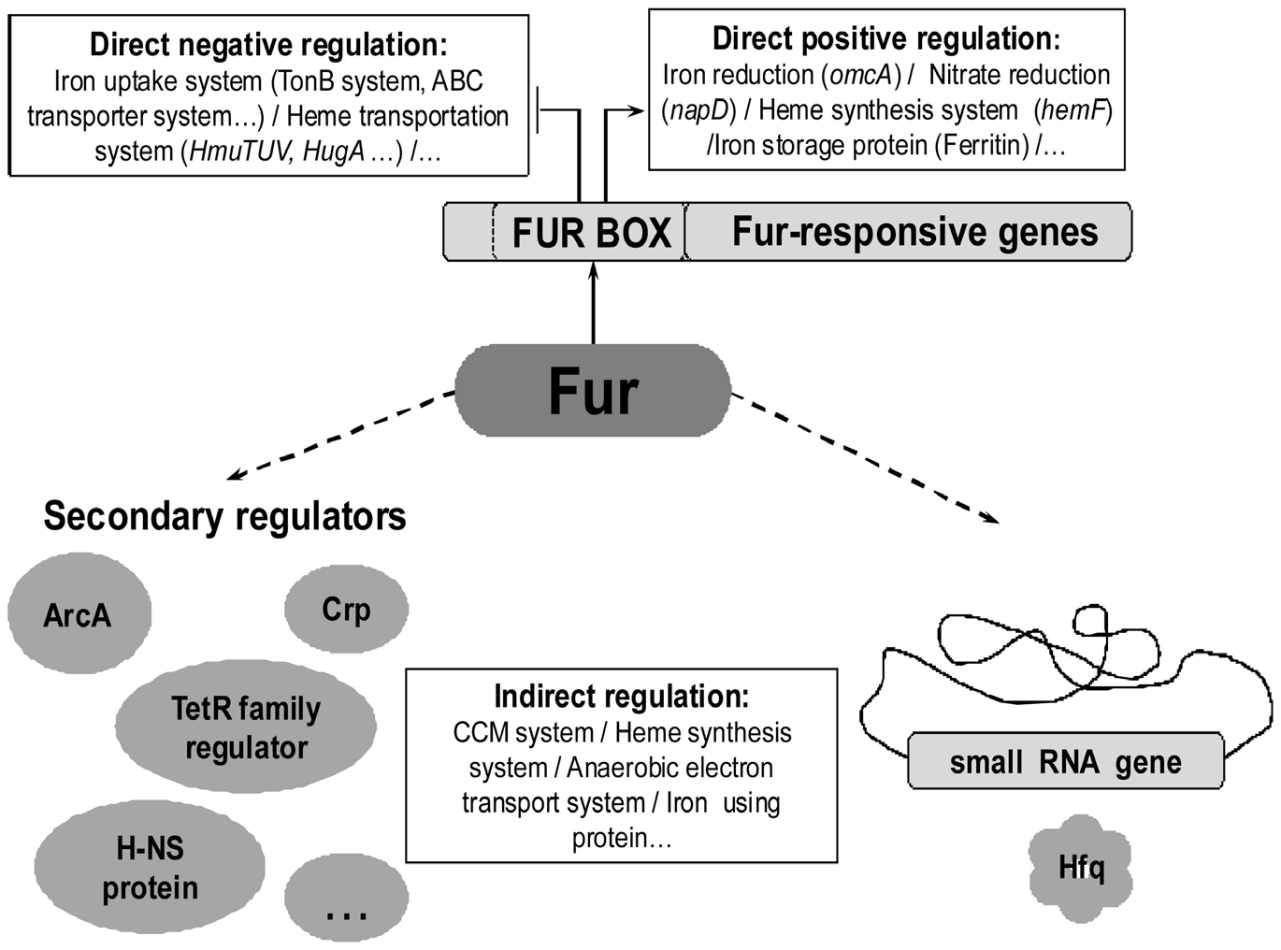
A model of the Fur regulatory system in WP3. In this model, Fur acts as both a direct and indirect regulator of iron homeostasis and anaerobic respiration. As a direct regulator, the Fur protein generally binds to a Fur Box region to down- or up-regulate the expression of genes. As an indirect regulator, the Fur protein represses an antisense non-coding regulatory RNA or controls secondary regulators, such as Crp, ArcA, the H-NS protein, and the TetR family regulator. These proteins are also capable of regulating genes in the anaerobic respiration system as well as those that encode iron using proteins. The genes regulated by Fur are involved in iron homeostasis, the anaerobic electron transport system, the heme biosynthesis and transport systems, and the CCM system. Direct regulation is depicted with a solid arrow, and indirect regulation is depicted with a dotted arrow.

Based on our results, we have proposed a model for Fur-related regulation of iron homeostasis and anaerobic respiration in WP3 (Figure 5). According to the model, Fur acts primarily as a negative regulator in the iron uptake system, where gene expression is regulated by the direct binding of Fur and Fur Box sequences. Alternatively, Fur could function as a positive regulator in the iron storage system. Fur regulates anaerobic respiration through various mechanisms. First, Fur may regulate anaerobic respiration indirectly by regulating the expression of secondary regulators such as the ArcA regulator, a transcriptional factor that is involved in aerobic/anaerobic respiration [53], [56], [67]; the TetR family transcriptional regulator [27]; the H-NS protein, which is involved in iron storage and virulence; and the Crp-like regulator. A conserved Fur Box was identified in the promoter region of the Crp-like regulator (as shown by EMSA), suggesting that Fur directly controls the expression of the Crp-like regulator gene. Notably, some genes in WP3 contain both Fur Box and Crp Box in their promoter regions. For example, the gene cluster involved in ferrous iron transport (swp3271-swp3270), gene cluster encoding fumarate reductase (swp0428-swp0431), and gene cluster involved in iron reduction (swp3272-swp3275). It is thus possible that dual regulatory role occurred between the Crp and Fur transcriptional regulators. The detail regulatory interplay between Fur and CRP warrants further investigation. Second, as reported for other bacteria [10], [50], non-coding small RNAs are involved in the regulation of iron-containing proteins, such as Fe-S proteins, that provide iron for central metabolic processes. Using MR-1 *ryhB* as a seed against the WP3 genome revealed a region of strong conservation, suggesting that this WP3 sequence may be a *ryhB*-like gene (5199865–5200032). The RNA chaperone Hfq was also identified in the WP3 genome (swp0789). Further studies are required to determine the role of Hfq in Fur regulation of anaerobic respiration in WP3. Third, Fur could directly regulate genes that are involved in anaerobic respiration, such as *napD* and *omcA*, and positively regulate genes that are closely related to anaerobic respiration, such as those involved in the heme biosynthesis system and the CCM system. Because only a small number of genes that are involved in anaerobic respiration possess upstream Fur Box motifs, indirect regulation likely plays a predominant role in the Fur regulation system.

In the deep-sea iron-reducing bacterium WP3, the anaerobic iron respiration pathway produces iron in ferrous form (Fe^2+^), which can be taken up easily by WP3 itself [33]. Under these conditions, WP3 has a stable iron source, and iron can be used as a stable environmental signal molecule. Oxygen has the highest redox potential of the EAs [68] and is consumed at a low level in deep-sea sediments. In such cases, sensitive sensors would be produced by microorganisms to detect oxygen concentrations. Oxygenation of ferrous iron occurs immediately in the presence of O_2_ inside of the cell, and the Fur-Fe^2+^ complex, which contains the remaining free Fe^2+^, is able to bind to the Fur-box. Therefore, the Fur protein appears to act as a sensor for anoxic conditions by responding to environmental redox changes and regulating various metabolic pathways, including the anaerobic respiration system. Previously, two different pathways (Fnr and ArcA) were known to control anaerobic metabolism and were associated with either the cellular oxidation or reduction (redox) status [69], [70]. The ability of Fur to function as a sensor of anoxic conditions in association with free Fe^2+^ suggests that the Fur sensor is more sensitive to and responds more rapidly to changes in the cellular redox status than to the cellular oxidation status, which is characterized by the [4Fe-4S] cluster or the oxidation state of membrane-bound quinines. In *Salmonella enterica*, Fur is involved in the control of nitrate/nitrite respiration by sensing the cellular redox status [17]. In summary, Fur could play important roles, such as an iron sensor, in response to environmental redox changes and in the regulation of various metabolic pathways, including anaerobic respiration.

## Supporting Information

Figure S1 **Anaerobic incubation of the WT WP3 and** ***fur*** **mutant strains on a CAS screening plate.**
(TIF)

Figure S2 **The identification of a predicted consensus of the Fur-binding motif in WP3 using the web-based tool RegPredict (** http://regpredict.lbl.gov **).** A sequence logo representation of a palindromic-motif model was derived based on those sites located upstream of the genes listed in Table S3. The error bars indicate the standard deviations of the sequence conservation.
(TIF)

Figure S3 **The heme biosynthesis pathway in WP3.** The pathway begins from L-Glutamate and proceeds through the formation of porphobilinogen, hydroxymethylbilane, and uroporphyrinogen III to coproporphyrinogen III, aided by five distinct enzymes (HemA-HemE). Next, HemF catalyzes the conversion of coproporphyrinogen III to protoporphyrinogen IX, and HemK catalyzes the subsequent formation of protoporphyrin IX. Lastly, heme is formed by HemH. The genes exhibiting attenuated expression in the *fur* mutant are highlighted in grey. Adapted from the KEGG database (http://www.genome.jp/kegg).
(TIF)

Figure S4 **Gene transcription levels of the 10 (CcmABCDEFGH, DsbA and DsbD) components involved in the cytochrome** ***c*** **maturation system in the WT WP3 and** ***fur*** **mutant strains under anaerobic conditions using fumarate as an EA.** The ATP-hydrolyzing CcmA subunits (swp2043) and their membrane integral partners CcmB (swp2042), CcmC (swp2041), and CcmD (swp2040) load heme onto the heme chaperone CcmE (swp2039). Meanwhile, apocytochrome c (apocyt c) translocates through the secretion system (signal sequence cleavage) and is oxidized by DsbA (swp2175). The electron transport complex (DsbD, swp 4520, and CcmG, swp2047) then reduces the disulfide bond of apocyt c. Lastly, the CcmF (swp2046) and CcmH (swp2048) complex ligate heme to apocyt c, and holocytochrome c is produced. The transcription level of WT WP3 was set as 1. The WP3 *pepN* gene was used to normalize the RNA concentration of each sample. The data shown represent 3 independent experiments, and the error bars indicate standard deviations.
(TIF)

Figure S5 **Fur binding to target promoters of** ***napD*** **,** ***omcA*** **and the Crp-like regulator gene.** Fur binding to the TonB receptor promoter and swp_1869 promoter (not predicted to be bound by Fur) were used as the positive and negative control, respectively. The DNA probe was pre-incubated with the purified Fur protein at the indicated molar ratios. The amount of DNA is 1 pmol, and 0, 4, 8, 16 pmol purified His tag fusion Fur were used in the DNA binding assays. The probes remained unbound in the absence of Fur binding, and reduced mobility was observed with increasing Fur concentration for all three of the Fur targets.
(TIF)

Table S1 Bacterial strains and plasmids used in the present study.
(PDF)

Table S2 Primers used in this study.
(PDF)

Table S3 Genes containing a putative Fur binding site in WP3.
(XLSX)
