# Supplementary figures and images for: The Regulatory Role of Ferric Uptake Regulator (Fur) during Anaerobic Respiration of Shewanella piezotolerans WP3
Source: PLoS One. 2013 Oct 4;8(10):e75588. doi: 10.1371/journal.pone.0075588 (PMC3790847; doi:10.1371/journal.pone.0075588)

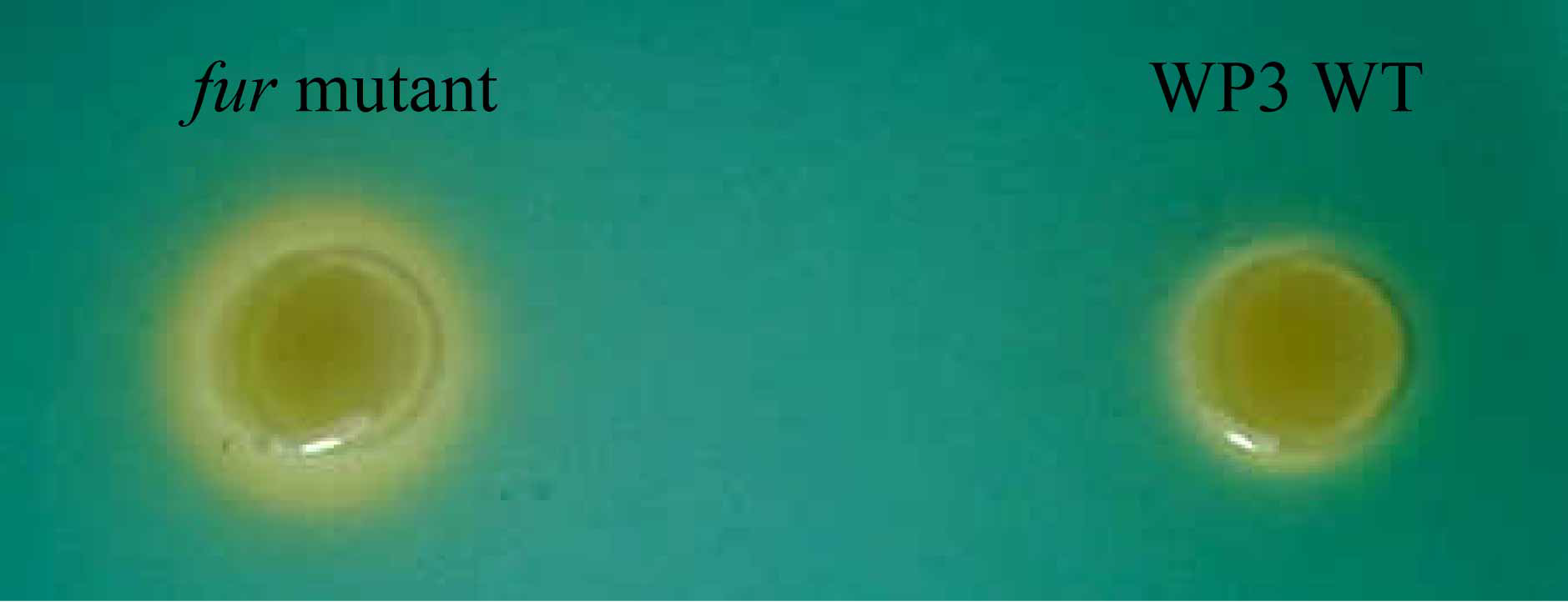

Supplement: Figure S1 — Anaerobic incubation of the WT WP3 and fur mutant strains on a CAS screening plate. (TIF) [file pone.0075588.s001.tif]

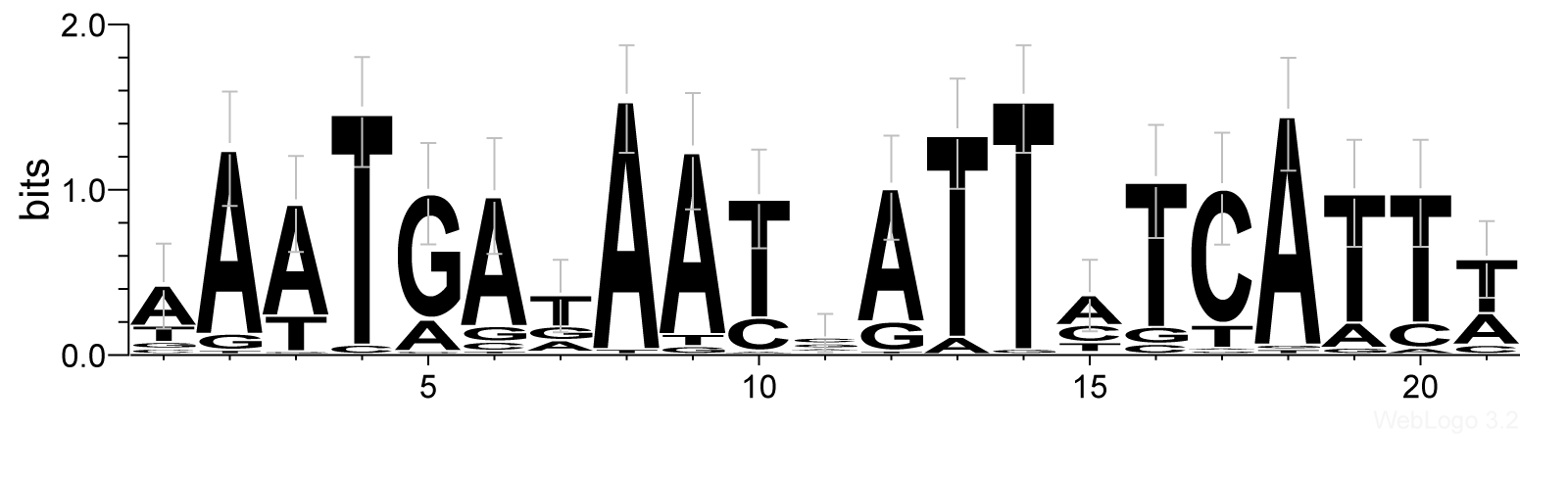

Supplement: Figure S2 — The identification of a predicted consensus of the Fur-binding motif in WP3 using the web-based tool RegPredict ( http://regpredict.lbl.gov ). A sequence logo representation of a palindromic-motif model was derived based on those sites located upstream of the genes listed in Table S3. The error bars indicate the standard deviations of the sequence conservation. (TIF) [file pone.0075588.s002.tif]

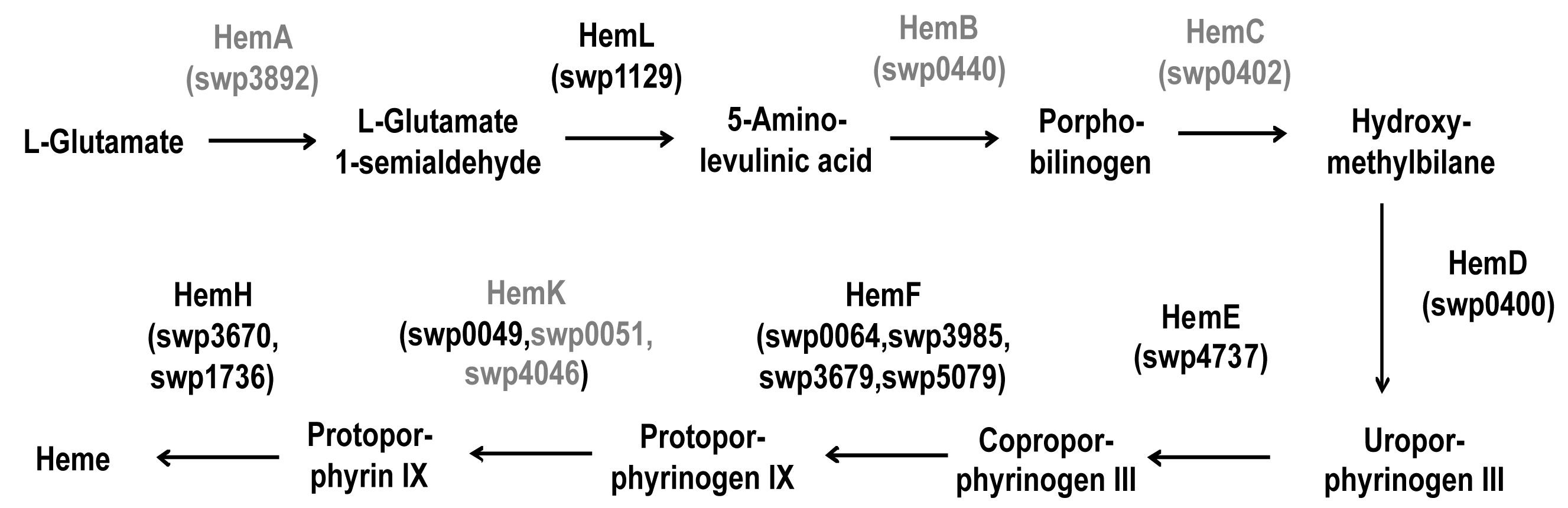

Supplement: Figure S3 — The heme biosynthesis pathway in WP3. The pathway begins from L-Glutamate and proceeds through the formation of porphobilinogen, hydroxymethylbilane, and uroporphyrinogen III to coproporphyrinogen III, aided by five distinct enzymes (HemA-HemE). Next, HemF catalyzes the conversion of coproporphyrinogen III to protoporphyrinogen IX, and HemK catalyzes the subsequent formation of protoporphyrin IX. Lastly, heme is formed by HemH. The genes exhibiting attenuated expression in the fur mutant are highlighted in grey. Adapted from the KEGG database (http://www.genome.jp/kegg). (TIF) [file pone.0075588.s003.tif]

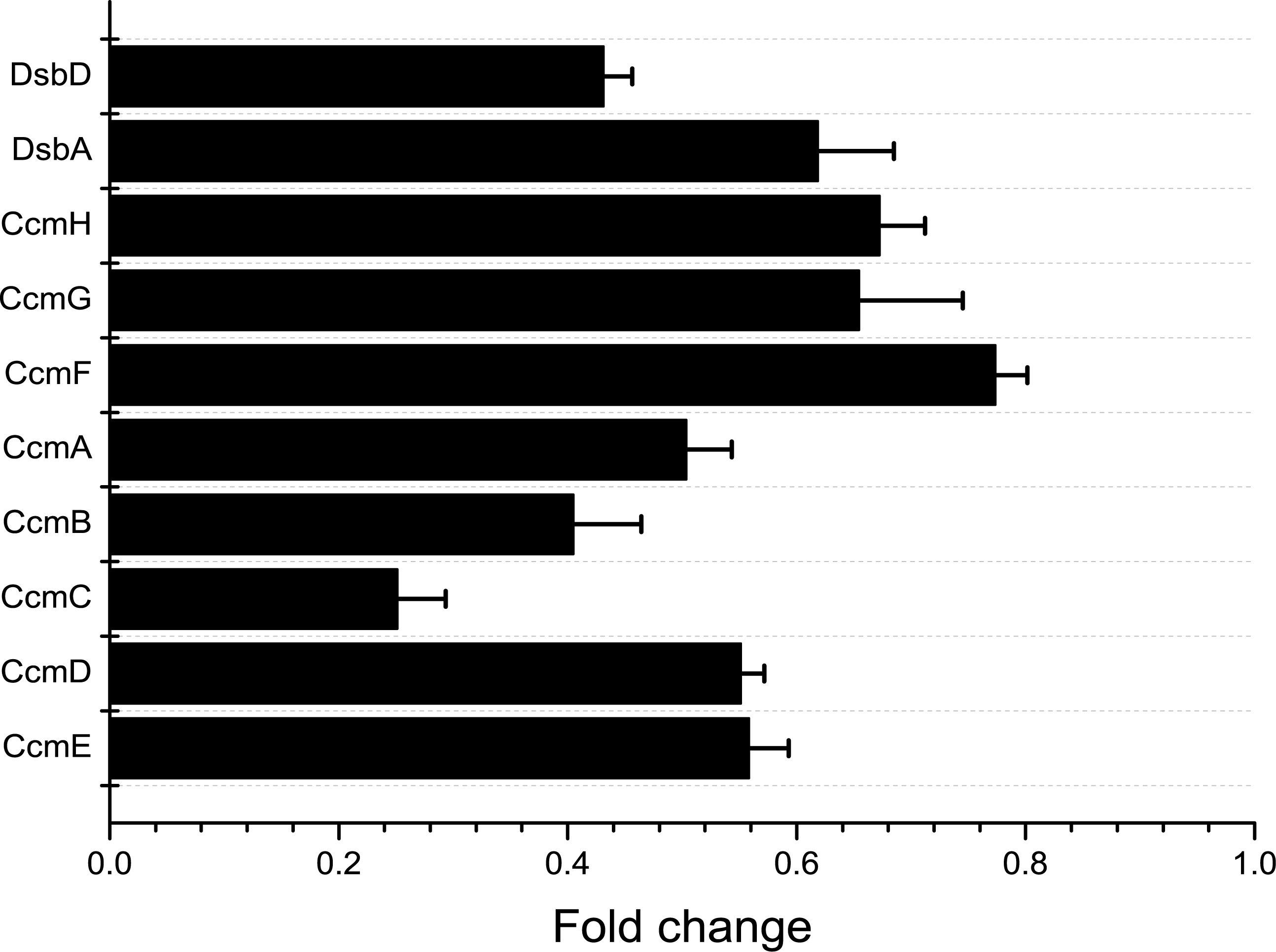

Supplement: Figure S4 — Gene transcription levels of the 10 (CcmABCDEFGH, DsbA and DsbD) components involved in the cytochrome c maturation system in the WT WP3 and fur mutant strains under anaerobic conditions using fumarate as an EA. The ATP-hydrolyzing CcmA subunits (swp2043) and their membrane integral partners CcmB (swp2042), CcmC (swp2041), and CcmD (swp2040) load heme onto the heme chaperone CcmE (swp2039). Meanwhile, apocytochrome c (apocyt c) translocates through the secretion system (signal sequence cleavage) and is oxidized by DsbA (swp2175). The electron transport complex (DsbD, swp 4520, and CcmG, swp2047) then reduces the disulfide bond of apocyt c. Lastly, the CcmF (swp2046) and CcmH (swp2048) complex ligate heme to apocyt c, and holocytochrome c is produced. The transcription level of WT WP3 was set as 1. The WP3 pepN gene was used to normalize the RNA concentration of each sample. The data shown represent 3 independent experiments, and the error bars indicate standard deviations. (TIF) [file pone.0075588.s004.tif]

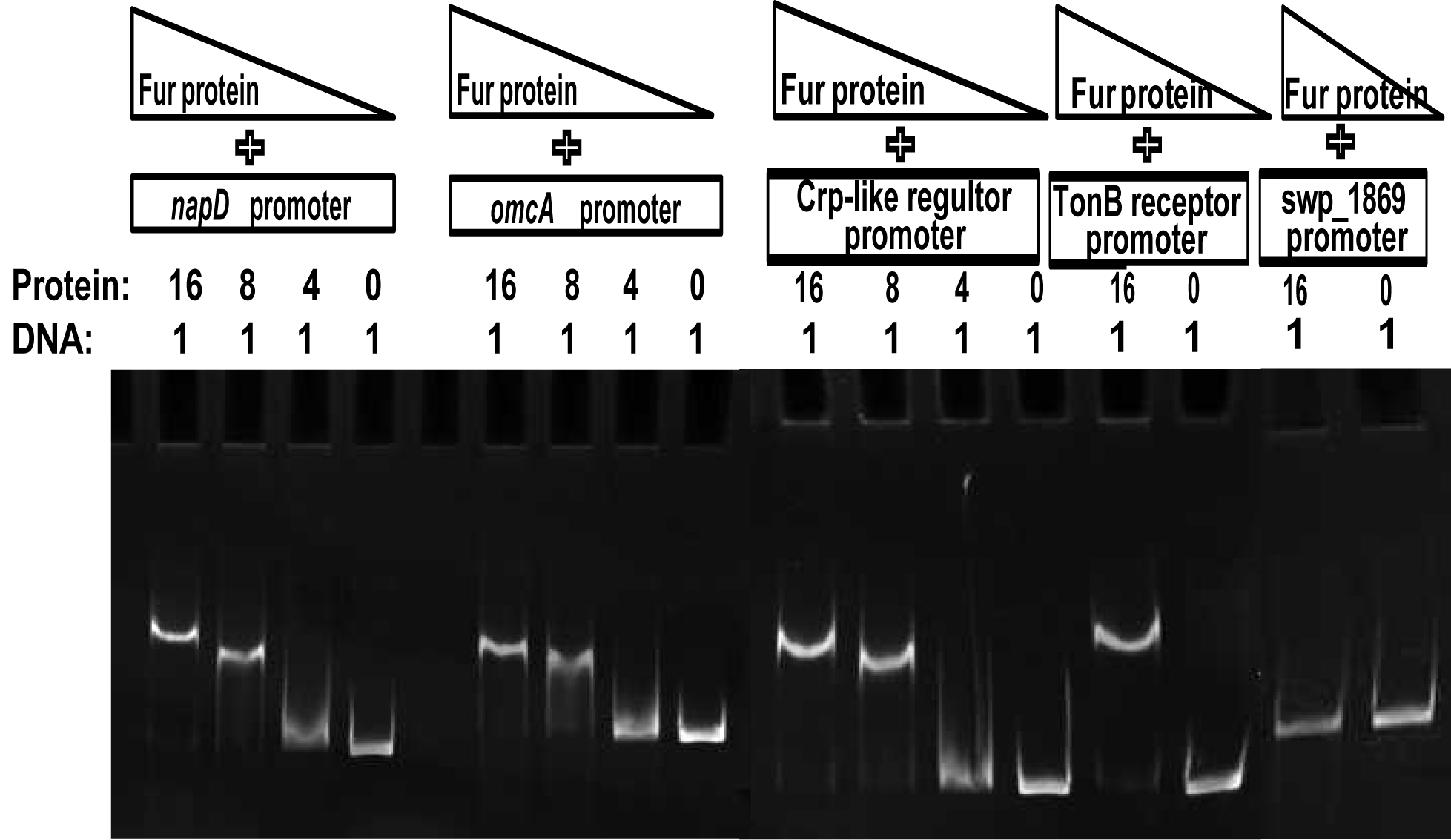

Supplement: Figure S5 — Fur binding to target promoters of napD , omcA and the Crp-like regulator gene. Fur binding to the TonB receptor promoter and swp_1869 promoter (not predicted to be bound by Fur) were used as the positive and negative control, respectively. The DNA probe was pre-incubated with the purified Fur protein at the indicated molar ratios. The amount of DNA is 1 pmol, and 0, 4, 8, 16 pmol purified His tag fusion Fur were used in the DNA binding assays. The probes remained unbound in the absence of Fur binding, and reduced mobility was observed with increasing Fur concentration for all three of the Fur targets. (TIF) [file pone.0075588.s005.tif]
