# Supplementary material for: The Regulatory Role of Ferric Uptake Regulator (Fur) during Anaerobic Respiration of Shewanella piezotolerans WP3
Source: PLoS One. 2013 Oct 4;8(10):e75588. doi: 10.1371/journal.pone.0075588 (PMC3790847; doi:10.1371/journal.pone.0075588)
Supplement: Table S1 — Bacterial strains and plasmids used in the present study. (PDF) [file pone.0075588.s006.pdf]

Table S1 Bacterial strains and plasmids used in the present study

Strains or plasmids	Description	Reference or source
E. coli strain		
WM3064	Donor strain for conjugation	Gao *et al*. (2006)
BL21 (DE3)	Protein expression strain	Lab stock
S. piezotolerans WP3 strains		
WT	Wild type	Lab stock
△*fur*	*fur* deletion mutant derived from WP3	This study
△*ccmC*	*ccmC* deletion mutant derived from WP3	This study
△*fccA*	*fccA* deletion mutant derived from WP3	This study
Plasmids		
pRE112	Allelic-exchange vector	Edwards *et al*. (1998)
pRE112-Fur	pRE112 containing the PCR fragment for deleting *fur*	This study
pRE112-CcmC	pRE112 containing the PCR fragment for deleting *ccmC*	This study
pRE112-FccA	pRE112 containing the PCR fragment for deleting *fccA*	This study
pSW2	Chl R, derived from the filamentous bacteriophage SW1; used for complementation	Unpublished work
pSW2-Fur	pSW1 containing *fur*	This study
pET28a	HIS-tag protein expression vector	Novagen
pET28a-Fur	pET28a containing *fur*	This study
