# Supplementary material for: The Regulatory Role of Ferric Uptake Regulator (Fur) during Anaerobic Respiration of Shewanella piezotolerans WP3
Source: PLoS One. 2013 Oct 4;8(10):e75588. doi: 10.1371/journal.pone.0075588 (PMC3790847; doi:10.1371/journal.pone.0075588)
Supplement: Table S2 — Primers used in this study. (PDF) [file pone.0075588.s007.pdf]

Table S2 Primers used in this study.

**Primers**	**Sequences** (5’-3’)
***fur* mutant**	
fur A Xba I	CTAGTCTAGACTGCTGCCAGCGCTCATTATAG
fur a	CATGCTCGCAGTTGTCTGTCAACGCGAGCCAGTTCATA
fur b	GACAACTGCGAGCATGGTGAG
fur B Sma I	TCCCCCGGGATATTACGCAGGCCGAAGAGATTT
***fur* complement**	
fur For Pst I	GAGACTGCAGATGACAGATGGAAATCAAGC
fur Rev Mlu I	TAAAACGCGTTTAAGGCTCACCATGCTCGC
***ccmC* mutant**	
ccmC A Sac I	AAGAGCTCGGGTTATCAAACTCTGCCGCAACTT
ccmC a	CTTCACCCGTACCGTCAACTCATGAGCAGCGCACCTTTGTGA
ccmC b	GGTACGGGTGAAGGCACGTTTTCCCACAGCTATCTTTTC
ccmC B Xba I	AATCTAGAAGGGCGGCTATATTCAGGCTATCAA
***fccA* mutant**	
fccA A Xba I	TAATTCTAGAGCCGTTTATGTTGCCTCAC
fccA a	ATAACTTACCTTCGGTAGAATGGCTGGT
fccA b	ACCGAAGGTAAGTTATCCGCAGCCATTG
fccA B Kpn I	TCCAGGTACCAGCCTTGATCGACACCGT
**RT-PCR**	
Heme maturation system	
swp2039 For	GGCGGCGAAATTATCGTAAC
swp2039 Rev	TGGCCCTCACGGAATAAATC
swp2040 For	GGGCGGCTATGCTTTTTATG
swp2040 Rev	GCGTTCCGAGAGAGAAGAAGGT
swp2041 For	GCGCTGTCATTACCTTTATTGCT
swp2041 Rev	GTGCCCCACATAGGCTTACC
swp2042 For	GGCCTGAGCCGCAAGTATTA
swp2042 Rev	AAGGAGTGCAGCTACCCAAATAA
swp2043 For	ATCACCGAAGGCGAGATTGT
swp2043 Rev	GACTGGTTTTTCCTGCACCATT
swp2046 For	CGGCGCTTGTTCACTCTCTT
swp2046 Rev	AACGGTCCAGTTACGGAATGC
swp2047 For	GCGACATGGTGTCCTTCATG
swp2047 Rev	GCGCGCCAACGTCATTA
swp2048 For	AGAGCTTTTCTGCGCTGGTATT
swp2048 Rev	TCAACCGGAGTCGCATTTACT
RNA-seq verification	
swp0429 For	GATGACATGGGTCGCCAAA
swp0429 Rev	CCATGACCGTCAGCCGATA
swp1055 For	CACTGTGACGCCGGAAGAA
swp1055 Rev	TGCATCTGCTCTGCAGCATT

swp1175 For	GCTTGGGAGAGCTTGATGAGA
swp1175 Rev	TCGGCATGCTTCATATCTTGAA
pepN For	TTAAGGCAATGGAAGCTGCAT
pepN Rev	CGTCTTTACCCGTTAATGATACGA
swp3209 For	GGTGAGTTCAACGGCAAAGG
swp3209 Rev	CGGTGTCATGGTACTCTTGTTTGT
swp3979 For	GCTGTGCTCGCCTCACAAA
swp3979 Rev	TGGCTGAGCCTGAATTAAGTTG
swp3980 For	GACTCTGCTGCTCAGTTATGCAA
swp3980 Rev	CACTTGGCAGCTCGCTTCTT
swp3981 For	TCGCCGCCGATAAAACA
swp3981 Rev	GCAGTGCCAACACATTCATCA
swp4950 For	CTGGCTGGGCGATCGA
swp4950 Rev	TCATCGTGTCAGCAGTCTTCGT
**Fur expression**	
Fur For EcoR I	GAGAGAATTCATGACAGATGGAAATCAAGC
Fur Rev XhoI I	TAAACTCGAGTTAAGGCTCACCATGCTC
**EMSA**	
EMSA_swp5150 For	AAGACGCCTGAAAAGACA
EMSA_swp5150 Rev	TTATTACGTGCAATAAGAAGC
EMSA_swp3277 For	TGACCTTAATCACATAAAGCAT
EMSA_swp3277 Rev	GCCACTACAGCCAGAAACT
EMSA_swp3806 For	TCGCTATCACGCTTATCC
EMSA_swp3806 Rev	CAGGCTGTAATTGACCTTTT
EMSA_swp4456 For	ATAGAGGGGAGCCATAACG
EMSA_swp4456 Rev	ATCGACCAAGATCAACTTTT
EMSA_swp3277 For	TGACCTTAATCACATAAAGCAT
EMSA_swp3277 Rev	GCCACTACAGCCAGAAACT
EMSA_swp1869 For	TGCCATACTTCCACTTCCTT
EMSA_swp1869 Rev	AGGCTGTTTTAACTCTGACT
